# Developmental Origins of Health and Disease

**DOI:** 10.1155/2012/838640

**Published:** 2012-05-15

**Authors:** Simon C. Langley-Evans, Barbara Alexander, Harry J. McArdle, Deborah M. Sloboda

**Affiliations:** ^1^School of Biosciences, University of Nottingham, Sutton Bonington, Loughborough LE12 5RD, UK; ^2^University of Mississippi Medical Center, 2500 North State Street, Jackson, MS 39216, USA; ^3^Rowett Institute of Nutrition and Health, University of Aberdeen, Greenburn Road, Bucksburn, Aberdeen AB24 3FX, UK; ^4^Faculty of Health Sciences, McMaster University, 1280 Main Street West, Hamilton, ON, Canada L8S 4L8

Risk of chronic disease during adulthood is partly determined by early life experience. Insults, both nutritional and nonnutritional, to the mother during pregnancy appear to programme physiological and metabolic function in the developing offspring, bringing about lasting changes to health. A robust body of epidemiological evidence shows that fetal exposure to maternal undernutrition, or maternal obesity, is associated with greater risk of coronary heart disease, type 2 diabetes, chronic renal disease, and a range of other conditions associated with ageing. The biological plausibility of this nutritional programming has been demonstrated by experiments in which the diet is manipulated during pregnancy in a wide range of animal species. The key focus for research in this area is to determine the mechanisms which lead from maternal insult to offspring disease phenotypes. 

In this special issue, R. J. Karp and colleagues report findings of a study of children aged 4–6 years, which demonstrated an association between body mass index in childhood and weight gain between the ages of 4 and 6 months. This work emphasises the importance of the relationship between early life influences, later adiposity, and the poor health associated with greater body fatness. This work is complemented by the animal study of J. Beltrand et al., which reports differences in weight gain and body fat in offspring of rats fed a high-fat diet compared to a control group. The mechanistic role of an exaggerated neonatal leptin surge in high-fat-exposed offspring is demonstrated by the ablation of postnatal weight gain following treatment with a leptin antagonist. Insults other than nutrition may also exert programming effects on the developing fetus. G. J. Kummet et al. considered the effects of exposing mouse fetuses to selective serotonin reuptake inhibitors. These drugs are widely prescribed to women of childbearing age and may complicate up to 10% of pregnancies. In mice, fetal exposure was found to generate a hypermetabolic state, associated with greater food intake, but lower body weight.

The mechanistic basis of early life programming is widely held to involve influences of nutrition or other maternal signals upon the epigenome. The epigenetic theory was explored by E. K. Zinkhan et al., who demonstrated that placental insufficiency in animals, a manipulation known to programme metabolic disturbance, was associated with alterations to methylation of histones associated with the IGF-1 gene. This link between the placenta and programming, either at the level of the epigenome, or by other mechanisms, may be explained by involvement in the endocrine crosstalk between mother and fetus. As reviewed by E. Aszatalos, transfer of glucocorticoids across the placenta may be a potent programming stimulus, regardless of nutritional status. S. Entringer et al. review the role of placentally derived estrogen in the setting of fetal adrenal cortical function, a physiological process which may have important consequences for homeostatic processes in adult life. 

The studies of K. J. P. Ryan et al. and S. Engeham et al. consider mechanistic issues that are further downstream from the initial programming insult and more closely associated with the observed disease phenotype. Both studies report findings from an established model of programming resulting from maternal protein restriction in the rat. In the context of renal programming, S. Engeham and colleagues report data which indicates a contribution of programmed mitochondrial function to later function. Protein restriction *in utero* lowers respiratory rate in mitochondria from adult kidneys. K. J. P. Ryan et al. examined the cardiac response to ischemia-reperfusion injury and noted that programming of *β*-adrenergic receptor expression may underpin some of the observed effects of prenatal protein restriction. 

*Simon C. Langley-Evans* *Simon C. Langley-Evans* *Barbara Alexander* *Barbara Alexander* *Harry J. McArdle* *Harry J. McArdle* *Deborah M. Sloboda* *Deborah M. Sloboda*

